# Long-term results of secondary intraocular lens implantation in children under 30 months of age

**DOI:** 10.1038/s41433-018-0191-3

**Published:** 2018-08-28

**Authors:** Camila R. Koch, Newton Kara-Junior, Alicia Serra, Marta Morales

**Affiliations:** 1 University of São Paulo (USP), São Paulo, Brazil; 2 Sant Joan de Déu Hospital, Barcelona, Spain

## Abstract

**Purpose:**

To report the long-term outcome of early secondary intraocular lens (IOL) implantation following congenital cataract extraction in a large number of eyes.

**Methods:**

Data of aphakic children under 30 months of age who underwent secondary IOL implantation and had at least one year of follow-up after the surgery was reviewed. In all of the patients, a foldable three-piece acrylic IOL was implanted in the ciliary sulcus by the same surgeon using the same technique. The database studied included refractive and visual acuity (VA) outcomes and complications.

**Results:**

Fifty patients (75 eyes) were included. The average age at the time of cataract extraction was 94.20 ± 44.94 days and 20.7 ± 6.0 months in the secondary IOL implantation. After 82.32 ± 48.91 months, the VA was 0.58 ± 0.35 LogMAR and the spherical equivalent was −2.20 ± 4.19 D. There was a negative correlation between a longer follow-up period and myopia at the SE measured (*P* = .001). The most frequent complications included glaucoma and corectopia. Performing the secondary IOL implantation ≤ 20 months of age was not a risk factor for glaucoma development (*P* = 0.095).

**Conclusion:**

Secondary IOL implantation under 30 months of age is an option for children with unsatisfactory management of the optic treatment. A predictable IOL power calculation and satisfactory visual outcomes compared to results of later secondary IOL implantation are possible.

## Introduction

Despite improvements in pediatric cataract surgery, the intraocular lens (IOL) power calculation is still the greatest challenge in young children. During the first six month of life, the increase in axial length and the change in corneal curvature are faster, making the selection of the IOL power and the prediction of myopia shift more accurate after this period [1–3]. Furthermore, it is recommended that children under seven months of age with unilateral cataract remain aphakic in order to avoid complications and additional surgeries [4]. On bilateral cataract cases, there is no consensus on the right time to perform primary IOL implantation [5]. Although IOLu2 study associates IOL implantation on bilateral cataract in children under two years of age to a potential improvement in visual acuity (VA), it also presents an increased reoperation rate while not reducing the risk of postoperative glaucoma [6].

Early correction of aphakia is the most critical component to prevent the development of amblyopia. It must be treated by having the child wear either contact lenses or glasses full-time whenever awake [7, 8]. Since the IOL provides a full-time partial correction and the management of the optical treatment becomes more difficult for parents as the child grows older, the IOL implant should be considered when the desired optical correction is no longer possible [9, 10]. Most research has shown satisfactory secondary IOL implant results in preschoolers [11–13], but the optimal time to perform it in aphakic children is uncertain.

This retrospective study shows the results in a long follow-up period of the secondary IOL implantation performed within 11 and 30 months of life in children who underwent congenital cataract surgery before the seventh month of life and were left aphakic with capsular support. The secondary IOL implantation was performed by the same surgeon and the follow-up was at least one year after the IOL implantation. The aim was to report the refractive changes, long-term visual outcomes, and complications in a large number of eyes in young children.

## Methods

### Study population

A retrospective review was performed on medical records at Sant Joan de Déu Hospital between 1 April 1999, and 31 May 2016, of children who underwent cataract surgery before seven months of age and were left aphakic. Data on children who received the secondary IOL implant before the age of 30 months were extracted from this database. The study followed the tenets of the Declaration of Helsinki and was approved by the Medical Institutional Review Board with the approval of Sant Joan de Déu Hospital, Barcelona, Spain.

### Data collection

Data collected included birth date, gender, laterality, patient age at the time of cataract surgery and secondary IOL implantation, the surgeon who performed the surgery, the type and power of the IOL implanted, axial length before the cataract surgery and before the secondary IOL implantation, the period between the cataract surgery and the IOL secondary implantation and from this time to the final follow-up, complications, refraction performed 30 days post-secondary implant, the corrected distance visual acuity (CDVA), and refraction at the final follow-up.

### Exclusion criteria

Exclusion criteria were secondary implant surgery performed by another surgeon, time of follow-up less than one year after the secondary IOL implantation, incomplete datasets, and surgery performed outside the pre-established time. Sixty-three patients (92 eyes) were identified in the data as having cataract surgery before seven months of life and secondary IOL implantation before 30 months of life during the study period. Thirteen patients (17 eyes) were excluded from analysis (three with incomplete postoperative datasets due to severe neurological delay, four had the secondary IOL implantation performed by another surgeon, and six did not complete at least one year of follow-up).

### Surgical technique

The selected patients underwent cataract surgery by two experienced surgeons (MMB and RPC) and the secondary IOL implantation surgery by a single surgeon (MMB). The operated eye was sterilized with half-strength povidone-iodine for two minutes in all of the surgical procedures. The steps of the cataract surgery in brief were anterior capsulotomy followed by aspiration of the lens, central posterior capsular opening, and vitrectomy via corneal approach. In all of the patients with indications for secondary IOL implantation, under general anesthesia and with dilated pupils, the retina, capsular support, corneal diameter, and IOL power were evaluated. The IOL implantation was performed in patients without changes in fundus examination, adequate capsular support, and with horizontal corneal diameter ≥ 11 mm. The keratometry measurement was performed using a handheld keratometer (CA KM-500, Nidek Inc., Fremont, CA, USA) and the AL was measured with contact biometry (Axis II A-scan, Quantel Medical, Paris, France) by the surgeon. The IOL power was calculated using the Sanders–Retzlaff–Kraff (SRK) II formula (before 2009) and SRK/T (after 2009), aiming at a hyperopic correction as a target refraction in the immediate postoperative period.

During the secondary IOL implantation surgery, a superior clear cornea incision of 3.2 mm was made and an ophthalmic viscosurgical device (OVD) was injected into the anterior chamber. If synechiae were present, lysis was performed followed by anterior vitrectomy and prior to IOL implantation in the ciliary sulcus. A three-piece foldable acrylic IOL was implanted in all of the children through the main incision with an injector. After the aspiration of OVD, the incision was sutured with 10-0 nylon and acetylcholine was injected into the anterior chamber. At the end of the procedure, an antibiotic was injected into the anterior chamber and an inferior subconjunctival injection of steroid was given. An antibiotic drop and a steroid ointment were used in the operated eye, which was then patched. The ciliary sulcus was the choice for site of IOL implantation. The surgical technique was the same in all patients.

### Postoperative assessment

Postoperatively, a topical combination of antibiotic and steroid drops were used every four hours for a week, followed by tapering the dose each week for four more weeks and cycloplegic eye drops twice daily for two weeks. Follow-up was performed one day, one week, 30 days, and every three months up to one year after the second IOL implantation, and thereafter every six months. The main outcome measures were postoperative complications, refraction changes, and CDVA at the final follow-up.

### Statistical analysis

Quantitative variables were expressed by mean and standard deviations. Qualitative variables were expressed by absolute and relative frequencies. The generalized estimating equation (GEE) method was used to compare the means. A *p*-value < 0.05 was considered statistically significant. The Mann–Whitey test was used to compare the mean between the SE according to the follow-up time (< and >30 months). The Statistical Package for the Social Sciences (SPSS, Inc., version 19.0) for windows software was used for statistical analysis.

## Results

### Baseline data

Fifty patients (75 eyes) were included. Forty-six (66.7%) had bilateral cataracts, 40 (53.3%) in the right eye, and 43 (57.3%) were male. Among the included eyes, 15 eyes in eight children had systemic diseases (three had Lowe syndrome, three had Down syndrome, one had cystic fibrosis, and one had autism). The most frequent cataract morphology was nuclear (42.7%), followed by total (25.3%) and lamellar (16.0%). The mean age of the cataract surgery was 94.20 ± 44.94 days (range 20–204) and 20.73 ± 6.02 months (range 11–30) at the secondary IOL implantation. The mean time elapsed between the cataract extraction and the secondary IOL implantation was 17.73 ± 5.87 months (range 7–28). The mean follow-up time was 82.32 ± 48.91 months (range 13–189).

### Visual and refractive outcomes

**Fig. 1:**
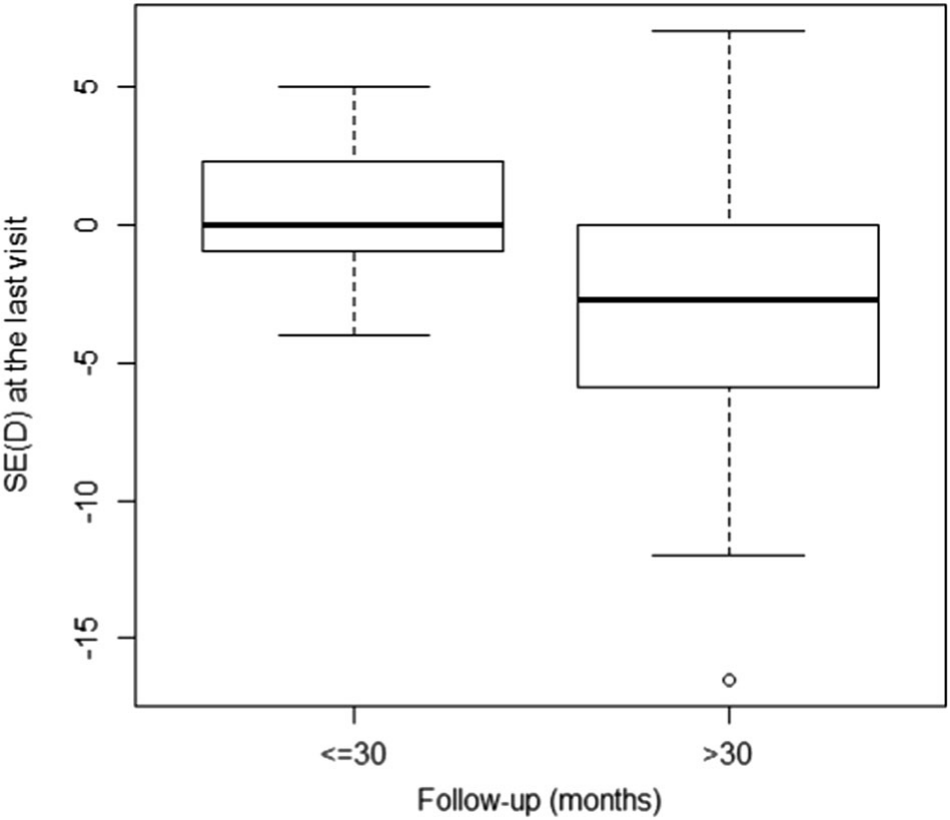
The box plot shows the spherical equivalent according to the follow-up time in months. SE spherical equivalent, D dioptre

Spherical equivalent (SE) 30 days after the IOL implantation averaged 2.16 ± 3.12 diopters (D) (range −4–8). At the last follow-up, the mean SE was −2.20 ± 4.19 D (range −16.50–7) and the corrected distance visual acuity (CDVA) was 0.58 ± 0.35 LogMAR (range 0.0–1.3). Among the 19 eyes with spherical equivalent up to −5.00 D at the last visit, five were glaucomatous eyes (mean SE −7.52 ± 2.64 D; range −5.25 to −16.50) and one eye had a retinal detachment. The mean SE when glaucomatous eyes were excluded, was −1.72 ± 3.66 D (range −8.25–7). The mean SE was 0.52 ± 2.52 in patients with less than 30 months of follow-up time and −2.80 ± 4.27 in patients with more than 30 months of follow-up, according to the Mann–Whitney test (*p* = 0.004). Fig. 1 shows the difference between the SE vs the follow-up time. Moreover, there was a negative correlation between a longer period of follow-up and myopia at the SE measured using Spearman’s correlation coefficient (*R* = −0.415, *p* = 0.001). The results according to the laterality are shown in Table 1. We included both eyes in the bilateral cataract cases because these presented different complications, CDVA and refractive error.

**Table 1:** Results according to laterality

Parameter	Bilateral (*n* = 48)		Unilateral (*n* = 27)		*P*-value
	Mean ± SD	Range	Mean ± SD	Range	
Age at cataract extraction (days)	96.42 ± 42.93	27, 186	89.76 ± 49.34	20, 204	0.603
Age at secondary IOL implantation (months)	21.58 ± 5.92	11, 30	19.04 ± 5.9	11, 30	0.124
AL at cataract surgery (mm)	18.15 ± 1.34	16.25, 22.24	17.58 ± 1.18	15.73, 20.00	0.110
AL at secondary IOL implantation (mm)	20.24 ± 1.43	17.71, 24.07	20.34 ± 1.57	17.76, 22.82	0.810
SE one month after IOL implantation (D)	2.07 ± 3.52	−4.00, 8.00	2.36 ± 2.21	−1.50, 6.50	0.723
SE at final follow-up (D)	−1.74 ± 3.74	−8.25, 7.00	−3.17 ± 4.96	−16.50, 4.25	0.242
Follow-up after secondary IOL implantation (months)	79.96 ± 51.42	14, 189	87.04 ± 44.07	13, 164	0.596
Age at last visit (months)	102.56 ± 56.69	35, 217	106.48 ± 48.04	29, 197	0.789
CDVA at last follow-up (LogMAR)	0.48 ± 0.27	0.0, 1.3	0.76 ± 0.42	0.1, 1.3	0.002
IOL power (D)	26.14 ± 3.77	17, 30	25.00 ± 3.67	14, 31	0.260

*CDVA* corrected distance visual acuity, *mm* millimetre, *D* dioptre, *IOL* intraocular lens, *SD* standard deviation, *AL* axial length, *logMAR*: logarithm of the minimum angle of resolution

### Intraoperative and postoperative complications

No intraoperative complications or intraoperative IOL issues were noted. The most implanted IOL type was MA60BM (AcrySof, Alcon, Fort Worth, TX, USA) in 62.7% of the patients, followed by HOYA PC-60AD (Hoya, Tokyo, Japan) in 13%, and Hydroview HP60M (Bausch & Lomb, Rochester, NY, USA) in 12%.

Of the 75 eyes, 12 (16%) had glaucoma developed. Table 2 shows the glaucoma complication details. Two of the eyes with open-angle glaucoma (OAG) were in one patient with Lowe syndrome. Two children with persistent fetal vasculature (PFV) were included in this study; both developed glaucoma. The three patients with OAG before the IOL implantation had undergone cataract surgery before two months of age (at 31, 54, and 58 days, respectively). Two of them needed tube implantation, one before the IOL implantation and the other after the IOL implantation. The mean time of the OAG diagnosis in the children after the IOL implantation was 50.17 ± 28.79 months (range 20–79). All surgical treatment in the OAG cases involved tube implantation. All of the angle-closure glaucoma (ACG) cases were controlled with peripheral iridectomy. One of these cases was an autism patient with IOL dislocation.

**Table 2:** Results according to glaucoma complications before and after secondary IOL implantation in 12 (16%) of 75 eyes

	Type of glaucoma		Age at cataract surgery	Age at IOL implantation	CDVA at last follow-up	Surgical
	OAG	ACG	(days)	(months)	(LogMAR)	cases
	(n%)	(n%)	Mean ± SD (range)	Mean ± SD (range)	Mean ± SD (range)	(n %)
Glaucoma (eyes)	9(75)	3(25)	84.58 ± 52.21 (27, 186)	22.58 ± 5.51 (12, 29)	0.93 ± 0.45 (0, 1.3)	7(58.33)
Before IOL implantation	3(25)	0(0)	47.67 ± 14.57 (31, 58)	22.33 ± 5.03 (17, 27)	1.23 ± 0.11 (1.1, 1.3)	2(16.66)
After IOL implantation	6(50)	3(25)	96.89 ± 54.90 (27, 186)	22.67 ± 5.93 (12, 29)	0.82 ± 0.48 (0, 1.3)	5(41.66)

*OAG* open-angle glaucoma, *ACG* angle-closure glaucoma, *IOL* intraocular lens, *SD* standard deviation

Corectopia was present in ten (13.33%) eyes, seven (70%) were after the IOL implantation. Five (50%) of these eyes underwent surgical repair. Visual axis opacity (VAO) was observed in six (8%) eyes. Of them, five eyes had VAO before IOL implantation (three needed surgery capsulotomy) and after the IOL implantation one had VAO (needed surgical capsulotomy). In these patients a vitrectomy probe via pars plicata was used to enlarge the capsulotomy. There was one IOL opacification (Hydroview HP60M IOL), one retinal detachment, and one endophthalmitis. The patient with retinal detachment needed a vitrectomy surgery. The patient with endophthalmitis was submitted to vitrectomy and IOL extraction. The overall complications related to surgery after the IOL implantation were in 19 eyes (25.33%). Of them, 13 (17.33%) had an additional surgery. Strabismus was found in 33 (64.7%) patients, being esotropia the most frequent in 18 (78.3%).

## Discussion

IOL provided a constant correction of refraction. To avoid amblyopia, the secondary IOL implantation was an option in cases in which the children were not doing well with contact lens correction. As per our hospital’s routine, children under seven months of life are left aphakic. In our department, when we realize that a child may not be undergoing the correct optical treatment and the parents complain about difficulty managing the use of contact lenses, we recommend secondary IOL implantation even before preschool age. Most studies [8, 14–17] reported later secondary IOL implantation in children. Earlier implantation with satisfactory results was reported by Kim et al. [18], with a mean age of 25.9 ± 3.9 months, followed by Rong et al. [19], with a mean age of 46.64 ± 29.37 months. To our knowledge, the results of our research are the earliest published. Despite satisfactory visual and refractive results obtained in most children, the high number of postoperative complications must be considered. The most common complication was glaucoma, but this might be due to the fact that these children underwent cataract extraction at an early age, and not to the secondary IOL implantation itself.

The final VA in this study corresponds to several results after congenital cataract surgery, VA being around 20/50 on bilateral cataract [7, 17, 18]. Among the children analyzed, 37.3% had better VA than 20/50 and 30.7% between 20/63 and 20/100. Strabismus was the most frequent complication not directly related to the surgical procedure, at 69.69% in the unilateral cataracts, and esotropia was the most frequent type of strabismus in our population. Given that strabismus is a frequent complication of very early congenital cataract surgery, particularly in unilateral cataracts, this result was expected [20, 21].

According to Rupal et al., axial length (AL) >23 mm is an important risk factor for developing IOL decentration or discolation [22]. In our study, all IOL implantation surgeries followed the same technique with an acrylic foldable three-piece IOL in the sulcus and were performed by a single surgeon. There was only one IOL dislocation, which caused an acute increase in intraocular pressure (IOP), which was resolved by repositioning the IOL. The mean AL of our children on the day of IOL implantation was 20.27 ± 1.46 mm, close to the result reported by Kim et al. (20.88 mm), who studied a population most similar to ours [18] and reported no IOL dislocation. These results are in agreement with Rupal et al., suggesting that smaller AL in young children avoids IOL decentration or dislocation. Wilson et al. used a non-foldable IOL in the sulcus to avoid IOL decentration [11]. However, our study showed that acrylic foldable IOL implantation in young children is secure and stable in the sulcus in a long follow-up. Moreover, only one child in our series had visual axis opacification (VAO) after the IOL implantation.

Glaucoma is the most serious complication following congenital cataract surgery. Aphakic glaucoma probably occurs due to damage and mechanical deformation of the support of the trabecular meshwork, either by inflammation or a toxic substance from the vitreous humour [23]. Early age at cataract surgery is a known risk factor for the development glaucoma [24]. In our series, 49.33% of the children had cataract surgery before three months of age. Among the complications, glaucoma most reduced VA, especially in the three cases of glaucoma in which the cataract was removed before two months of age. One of them needed tube implantation after the secondary IOL implantation to control the IOP, and the IOP of the other two continued to be controlled by medication. Trivedi et al. found that preoperative aphakic glaucoma is a risk factor for increased IOP following secondary IOL implantation. We also recommend more attention in these cases during the postoperative phase of the IOL implantation. Another risk factor for the development of glaucoma are patients with Lowe syndrome [25]. In our series, 2/6 eyes with Lowe syndrome had glaucoma, however, it was less than expected when compared to the literature.

The literature results of postoperative glaucoma on later secondary IOL implantation ranged from 0 to 32% [8, 17, 18]. Due to different surgical techniques and variable follow-up times, it is difficult to compare to our results. Many of these studies do not have a long follow-up time that would affect their results since glaucoma could happen at a later period not covered in their time frame. In our study, we had a long follow-up period (82.32 months) and all cases of open-angle-glaucoma that occurred after the IOL implantation (six eyes) were diagnosed at least 20 months after this surgery. Therefore, we recommend a long follow-up after IOL implantation in children. A possible reason for these late cases of glaucoma is the placement of the IOL in the sulcus, described as a probable risk factor compared to an implant in the capsular bag [11], however, it is not our practice to open the Soemmerring’s ring to attempt in-the-bag. The attempt to open the ring could be an additional risk for the eye since this technique requires cleaning all the grown cells within the Soemmerring’s ring. During this procedure the vitreous is beside or below the ring, and it might be caught. Moreover, this technique might damage the zonula.

We know that axial length growth is incredibly rapid in the first six months of life and slower during infancy and childhood [26, 27]. Therefore, after 11 months it is possible to perform a better calculation of the IOL power. The aim of a hyperopic correction as a target refraction in the immediate postoperative period was achieved in most of the patients and the predictability of the refractive error had a reasonable rate (spherical equivalent (SE) −2.20 ± 4.19 D at the last visit). The negative and fair Spearman’s correlation showed that the longer the follow-up period of our children, the more myopic cases would occur, albeit with a low myopic SE, but it would have been more accurate if the effective lens position (ELP) had been made during the IOL power calculation since we implanted it in the ciliary sulcus. Furthermore, before 2009, the SRK II formula was used, which is no longer the preferred IOL power calculation formula. SE was more myopic in children who were treated using the SRK II formula than the SRK/T formula (SE −4.06 D vs −0.21 D, *p* < 0.001) at the last follow-up time. These results are in accordance with O’Gallagher et al. [28]. It suggests that with the ELP calculation and the new formula, it is possible to perform an adequate predictive refraction in secondary IOL implantation in the ciliary sulcus of young children.

This study was limited by its retrospective design, by the fact that more than one type of IOL was implanted, and by the fact that the ELP was not calculated. In conclusion, our results suggest that it is an option to perform the secondary IOL implantation after congenital cataract surgery at an early age, especially for those who did not adapt well to contact lenses. It is possible to reach a good predictability of the refractive error and a more accurate calculation of the IOL power with newer generation IOLs and more recent formulas. More studies with an updated IOL formula calculation in a long-term follow-up are necessary.

### Summary

#### What was known before

- Secondary IOL implantation at preschool age is a safe procedure and also offers an accurate IOL power calculation.
- Reaching a predictability of the refractive error is still a challenge in IOL implantation in young children.

#### What this study adds

- A satisfactory IOL power calculation is possible before preschool age and a three-piece IOL implantation in ciliary sulcus after cataract extraction is safe in a long term.
- Secondary IOL implantation in children under thirty months of age is an option for patients with unsatisfactory management of the optic treatment.
